# Corilagin alleviates intestinal ischemia/reperfusion-induced intestinal and lung injury in mice *via* inhibiting NLRP3 inflammasome activation and pyroptosis

**DOI:** 10.3389/fphar.2022.1060104

**Published:** 2022-11-23

**Authors:** Wenlian Li, Kejia Yang, Bin Li, Yunxiang Wang, Jing Liu, Dapeng Chen, Yunpeng Diao

**Affiliations:** ^1^ College of Pharmacy, Dalian Medical University, Dalian, China; ^2^ Dalian Anti-Infective Traditional Chinese Medicine Development Engineering Technology Research Center, Dalian, China; ^3^ Comparative Medicine Department of Researching and Teaching, Dalian Medical University, Dalian, China

**Keywords:** corilagin, ischemia-reperfusion, intestine, lung, NLRP3, pyroptosis

## Abstract

Intestinal ischemia reperfusion (II/R) is a clinical emergency that frequently occurs in a variety of clinical conditions. Severe intestinal injury results in the release of cytotoxic substances and inflammatory mediators which can activate local inflammatory response and bacterial translocation. This triggers multi-organ failure, including lung injury, which is a common complication of II/R injury and contributes to the high mortality rate. Corilagin (Cor) is a natural ellagitannin found in a variety of plants. It has many biological and pharmacological properties, including antioxidant, anti-inflammatory and anti-apoptosis activities. However, no studies have evaluated the effects and molecular mechanisms of Cor in alleviating II/R-induced intestinal and lung damage. In this study, Cor was found to significantly alleviate II/R-induced pathological damage, inflammatory response, oxidative stress, NLRP3 inflammasome activation, and pyroptosis in intestinal and lung tissues both *in vivo* and *in vitro*. Further, Cor inhibited the NLRP3 inflammasome activation and pyroptosis in RAW264.7 and MLE-12 cells induced by LPS/nigericin and that in IEC-6 cells induced by nigericin, indicating an amelioration of Cor in II/R-induced intestinal and lung injury *via* inhibiting NLRP3 inflammasome activation and pyroptosis. Thus, Cor might be a potential therapeutic agent for II/R-induced inflammation and tissue injury.

## Introduction

Ischemia-reperfusion (I/R) injury occurs when the blood supply is temporarily interrupted and leads to organ damage or even organ failure. Intestinal I/R (II/R) injury is a life-threatening surgical emergency that usually occurs during clinical localized interventions or as a severe complication of some systematic diseases, such as severe infection, traumatic shock, and cardiopulmonary disease (Lehtimäki et al., 2015). The pathogenesis of II/R injury is multi-factorial and is associated with excessive inflammatory cytokines release, oxidative stress and epithelial cell death, leading to impaired intestinal barrier function with increased intestinal permeability and intestinal flora translocation, which will further result in severe local and systemic inflammation and multiple organ dysfunction syndrome (Hu et al., 2018). In particular, the lung is one of the most sensitive remote organs to II/R and is susceptible to developing acute injury because of excessive inflammatory response. Therefore, there is a critical need for innovative therapeutic strategies to ameliorate II/R-induced intestinal and lung injuries.

Increasing evidence has shown that NOD-, LRR- and pyrin domain-containing 3 (NLRP3) inflammasome plays crucial roles in the development of various diseases by initiating inflammatory responses (Rathinam and Fitzgerald, 2016). When under several stress conditions, especially oxidative stress, NLRP3 inflammasome components are assembled to activate caspase-1 with subsequent maturation of IL-1β and GSDMD, resulting in inflammation and pyroptosis. Pyroptosis is a newly discovered programmed cell death process that frequently occurs in various organs and tissues (Kovacs and Miao, 2017; Ito et al., 2020; Jia et al., 2020). Besides cell death, pyroptosis also causes excessive inflammatory damage. Recently, NLRP3-related pyroptosis has been shown to play important roles in multiple I/R injuries in heart, liver, kidney, intestine, and lung by mediating the initial inflammatory response and subsequent inflammatory cell death (Qiu et al., 2017; Ito et al., 2020; Jia et al., 2020; Zhang et al., 2020; El-Sisi et al., 2021; Ni et al., 2021). Inhibition of NLRP3 inflammasome activation protects against II/R-induced intestinal and lung injury (Jia et al., 2020; Zhang et al., 2020). Therefore, NLRP3 inflammasome activation and the resulting pyroptosis plays fundamental roles in the severity of II/R injury.

**FIGURE 1:**
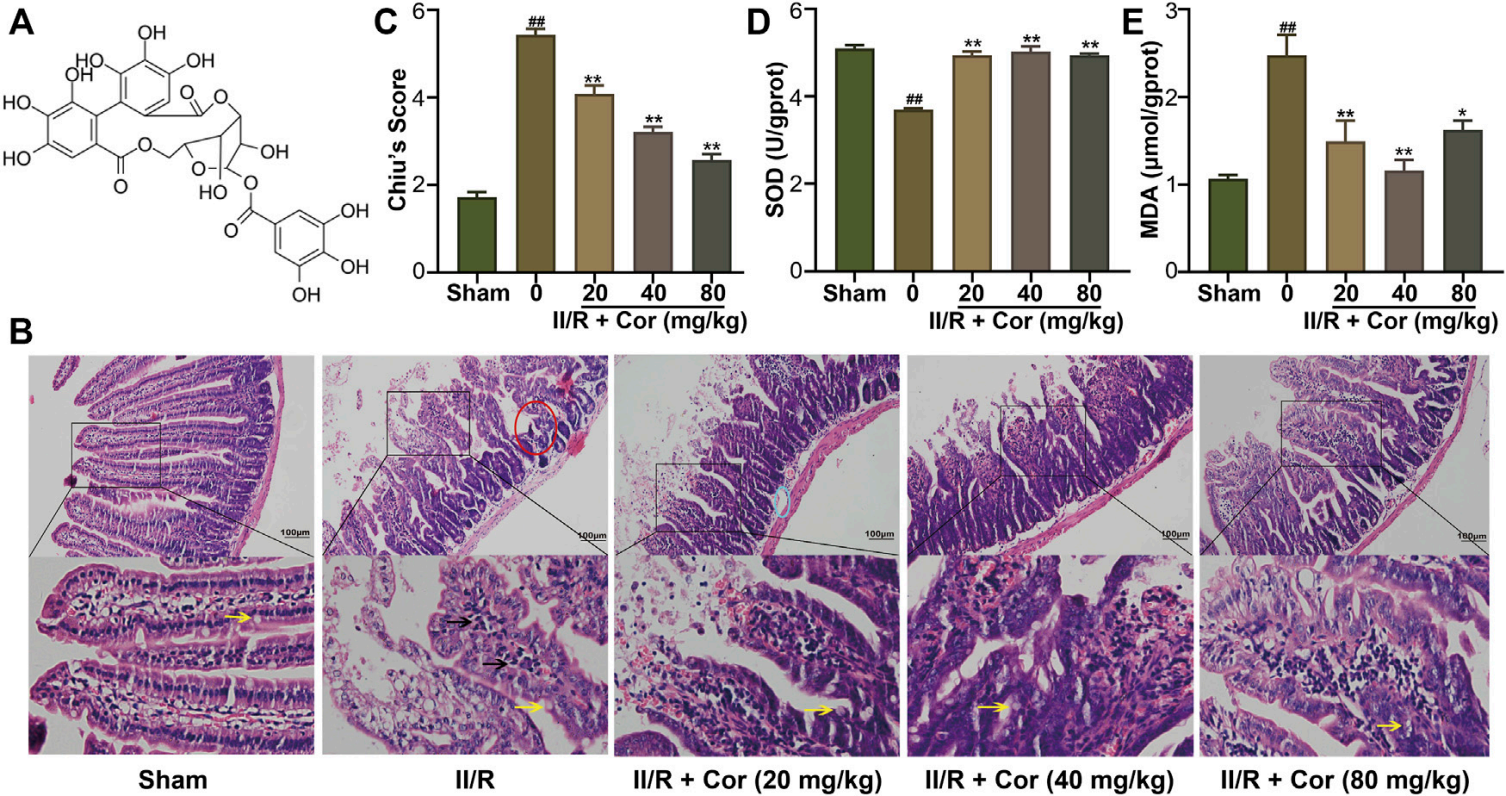
Cor ameliorated II/R-induced intestinal damage in mice. **(A)** The chemical structure of Cor. **(B)** Representative images of intestinal histology (scale bar = 100 μm). Goblet cells and inflammatory cells infiltration were indicated by arrows with yellow and black colors, respectively. **(C)** Histopathological scores (Chiu’s score) of the intestine. **(D,E)** Effects of Cor on SOD and MDA in intestines. Data are expressed as the mean ± SEM (n≥3), ^##^ *p* < 0.01 vs. sham group, *p < 0.05 and **p < 0.01 vs. II/R group.

Corilagin (Cor, Figure 1A), a natural ellagitannin found in a variety of plants, has been shown to have many biological and pharmacological properties such as antioxidant, anti-inflammatory, anti-apoptosis, anti-bacterial, anti-diabetic, and anti-tumor activities (Jin et al., 2013; Ding et al., 2017; Guo et al., 2017; Gupta et al., 2019). Cor has also been reported to improve ischemic brain injury in rats by reducing oxidative stress and promoting angiogenesis and protect against I/R-induced acute lung injury by improving apoptotic pathways (Ding et al., 2017; Guo et al., 2017). However, no studies have evaluated the effects and molecular mechanisms of Cor in alleviating II/R-induced intestinal and lung damage. Due to its excellent antioxidant and anti-inflammatory properties, we proposed that Cor might effectively ameliorate II/R injury by inhibiting II/R-induced oxidative stress and excessive inflammatory response, and the resulting inflammatory cell death. Thus, the aim of this study was to clarify the protective effect of Cor in II/R-induced intestinal and lung injury and to demonstrate the underlying mechanism.

## Materials and methods

### Chemicals and materials

Corilagin (Cor, purity ≥ 98%) was purchased from Shanghai Yuanye Bio-Technology Co., Ltd. (B20672, Shanghai, China), and prepared at appropriate concentrations in normal saline and serum/glucose-free DMEM medium for *in vivo* and *in vitro* experiments, respectively. Anti-NLRP3 (19771-1-AP), anti-Caspase1 P20 (22915-1-AP), anti-ASC (10500-1-AP), anti-Bax (50599-2-Ig), anti-Bcl-2 (12789-1-AP), anti-Caspase-3 (19677-1-AP) and anti-β-actin (20536-1-AP) were obtained from Proteintech Group (Wuhan, China). Anti-GSDMD (ab219800) was brought from Abcam (Boston, United States). Cell counting kit-8 (CCK-8, HY-K0301), Lipopolysaccharides (LPS, HY-D1056) and Nigericin (28643-80-3) were bought from MedChemExpress (Shanghai, China). Reagent kits for detecting superoxide dismutase (SOD, A001-3-2), glutathione (GSH, A006-2-1), malondialdehyde (MDA, A003-1-2), lactate dehydrogenase (LDH, A020-2-2) and myeloperoxidase (MPO, A044-1-1) were obtained from Nanjing Jiancheng Institute of Biotechnology (Nanjing, China). ELISA kits for measurement of tumor necrosis factor alpha (TNF-α, PT512) and interleukin-1β (IL-1β, PI301) were supplied by Beyotime Biotechnology (Shanghai, China). Dulbecco’s minimum essential medium (DMEM, 11960044), nonessential amino acids, Fetal bovine serum (FBS, FND500) and glutamine were purchased from Gibco (CA, United States). Protein extraction kits and bicinchoninic acid protein assay kits (BCA, DQ111-01) were obtained from TransGen Biotech (Beijing, China). Rat small intestinal crypt epithelial cell line (IEC6), mouse macrophage cell line (RAW264.7) and mouse lung epithelial cell line (MLE-12) were purchased from American Type Culture Collection (ATCC). FITC (GB22403), DAPI (G1012), Hoechst 33342/PI (CA1120), triton-100X (G5060) and bovine serum albumin (BSA, G5001) were provided by Serviobio (Wuhan, China).

### Animals

Male C57BL/6J mice (8 weeks old, 18–22 g) were provided by Experimental Animal Center of Dalian Medical University (Certificate of Conformity: No. SYXK (Liao) 2018-0001). The experimental protocol was approved by the Animal Care and Ethics Committee of Dalian Medical University (approval number: AEE19007) and performed according to the Principles for the Care and Use of Laboratory Animals in Research (State Council of China, 1988). All mice were housed for 14 days under optimal conditions of 25°C, 50% humidity, 12/12 day-night cycle and free access to food and water prior to the experiment.

The mouse II/R model was established as previously described (Wen et al., 2019). Mice were fasted for 24 h with free access to water before the experiments. After being anesthetized with sodium pentobarbital (50 mg/kg), the superior mesenteric artery (SMA) was clamped with a noninvasive microvascular clamp for 45 min, and the clamp was then gently released for 2 h reperfusion.

Mice were randomly assigned into five groups (*n* = 6). 1) Sham group: normal saline was administered *via* intragastric gavage for 3 days before sham surgery; 2) II/R group: animals were subjected II/R model after being given a normal saline *via* intragastric gavage for three consecutive days; 3) II/R + Cor (20 mg/kg) group: animals were pretreated with Cor (20 mg/kg, ig) once daily for 3 days before the II/R surgery; 4) II/R + Cor (40 mg/kg) group: animals were pretreated with Cor (40 mg/kg, ig) once daily for 3 days before the II/R surgery; and 5) II/R + Cor (80 mg/kg) group: animals were pretreated with Cor (80 mg/kg, ig) once daily for 3 days before the II/R surgery. After II/R reperfusion, executed the mice, and collected intestinal and lung tissues. Parts of the isolated intestinal and lung tissues were fixed in 4% paraformaldehyde solution for 24 h, and then used for histological analysis. The remaining parts of intestinal and lung tissues were flushed rapidly with ice-cold normal saline and immediately stored in liquid nitrogen for biochemical analysis and Western blot analysis later as previously described (Wen et al., 2019).

### Biochemical analysis

The intestinal and lung tissues were homogenized in ice-cold normal saline. And the homogenates were subsequently centrifuged at 3,500 g/min at 4°C for 20 min. The supernatant of intestinal and lung homogenates was used to measure the SOD and MPO, and the content of MDA, GSH, TNF-α and IL-1β by using the commercial test kits as instructed.

### Cells

IEC-6 and RAW264.7 cell lines were grown in DMEM supplemented with 10% FBS, 50 U/ml penicillin and 50 U/ml streptomycin. MLE-12 was cultured in DMEM/F-12 supplemented with 10% FBS, 50 U/ml penicillin and 50 U/mL streptomycin. All the cells were grown at 37°C and in a humidified atmosphere containing 5% CO_2_. We established oxygen-glucose deprivation/reoxygenation (OGD/R) to mimic II/R injury *in vitro*, cells were incubated in serum/glucose-free DMEM in a 5% CO_2_, 1% O_2_ and 94% N2 hypoxic chamber at 37°C to simulate ischemia by deprivation of oxygen and glucose. After incubation for 3 and 1 h, cells were cultured in normal DMEM supplemented with 10% FBS under normoxic conditions at 37°C for 12 and 1 h respectively for MLE-12 and IEC-6 cells to simulate reperfusion. RAW264.7 cells were treated with LPS (100 ng/ml) for 3 h and followed with Nigericin (10 μM) for another 4 h to active NLRP3 as previously described (Zhao et al., 2021a). The cell viability was determined by CCK-8 kits according to the manufacture’s suggestions. To assess pyroptosis, LDH leakage, Hoechst33342/PI double stain and ROS analysis was performed according to the manufacture’s suggestions.

### Immunofluorescence

Immunofluorescence analysis was performed as previously reported (Ye et al., 2021). RAW264.7, MLE-12 cells were placed in 24-well plates overnight. After pre-treatment with Cor (40 μM) for 24 h, LPS (100 ng/ml) for 3 h and then Nigericin (10 μM) for 4 h, the cells were fixed with 4% paraformaldehyde and permeabilized with 0.1% Triton X-100. After BSA closure, the cells were incubated overnight with anti-ASC antibody and then with FITC-conjugated donkey anti-rabbit IgG for 1 h. After staining with DAPI solution (10 mg/ml phosphate-buffered saline) to show the nuclei, the cells were observed under Leica TCS SP5II confocal microscope (Leica Microsystems, Wetzlar, Germany).

### Western blot analysis

Total proteins of intestine and lung tissues were extracted with RIPA lysate and subsequently centrifuged at 12,000 g for 15 min. After determination of protein concentration by BCA method, proteins were separated by sodium dodecyl sulfate-polyacrylamide gel electrophoresis (SDS-PAGE). The proteins were then transferred to polyvinylidene difluoride (PVDF) membranes. The membranes were closed with 5% skim milk and incubated with anti-NLRP3, anti-Caspase1 P20, anti-ASC, anti-GSDMD, anti-Bax, anti-Bcl-2, anti-cleaved (cl)-Caspase 3, GAPDH and anti-β-actin overnight at 4°C at 1:1000 ratio. Then, the bolts were incubated with horseradish peroxidase-conjugated secondary antibodies (1: 10000; Abcam, United States) for 2 h at 37°C. The expressions of proteins were normalized to β-actin or GAPDH. Using ImageJ to count the grayscale values of protein bands. Calculate the ratio of the destination bands to β-actin or GAPDH.

### Statistical analysis

The animal experiments, *in vitro* experiments, and data analyses were conducted according to a single-blind study design. One-way ANOVA analysis of variance with Tukey’s multiple comparisons test as post-test were used to determine statistical significance of results. All experiments were repeated at least three times and *p* < 0.05 was considered as statistically significant.

## Results

### Cor ameliorated II/R-induced intestinal injury

Hematoxylin-eosin (H&E) staining analysis showed that the jejunal mucosal epithelium in the sham group was intact with clearly visible goblet cells (yellow arrow). Compared with the sham mice, II/R induced significant intestinal morphological changes with massive inflammatory cell infiltration (black arrow) accompanied by denuded villi (red circle), submucosal edema (blue circle) and digestion as well as disintegration of lamina propria, hemorrhage, and ulceration. The II/R-induced intestinal morphologic alterations were significantly attenuated by pre-treatment with Cor (20, 40 and 80 mg/kg) in a dose-dependent manner with Chiu’s score markedly restored from 5.43 ± 0.14 to 2.57 ± 0.14 (*p* < 0.01, Figures 1B,C). The histopathological analysis suggested the protective effects of Cor pre-treatment against II/R-induced intestinal epithelial injury.

To characterize intestinal oxidative stress, the SOD activity and MDA content were determined. The SOD activity was significantly decreased, and the MDA content was markedly increased in II/R mice compared with the sham group. Cor (20, 40 and 80 mg/kg) pre-treatment not only reversed the decreased SOD activity from 3.69 ± 0.03 to 4.93 ± 0.04 U/gprot (*p* < 0.01), but also reversed the increased MDA content from 2.47 ± 0.24 to 1.62 ± 0.11 μmol/gprot (*p* < 0.05), as compared with II/R mice (Figures 1D,E).

### Cor ameliorated II/R-induced lung injury

**FIGURE 2:**
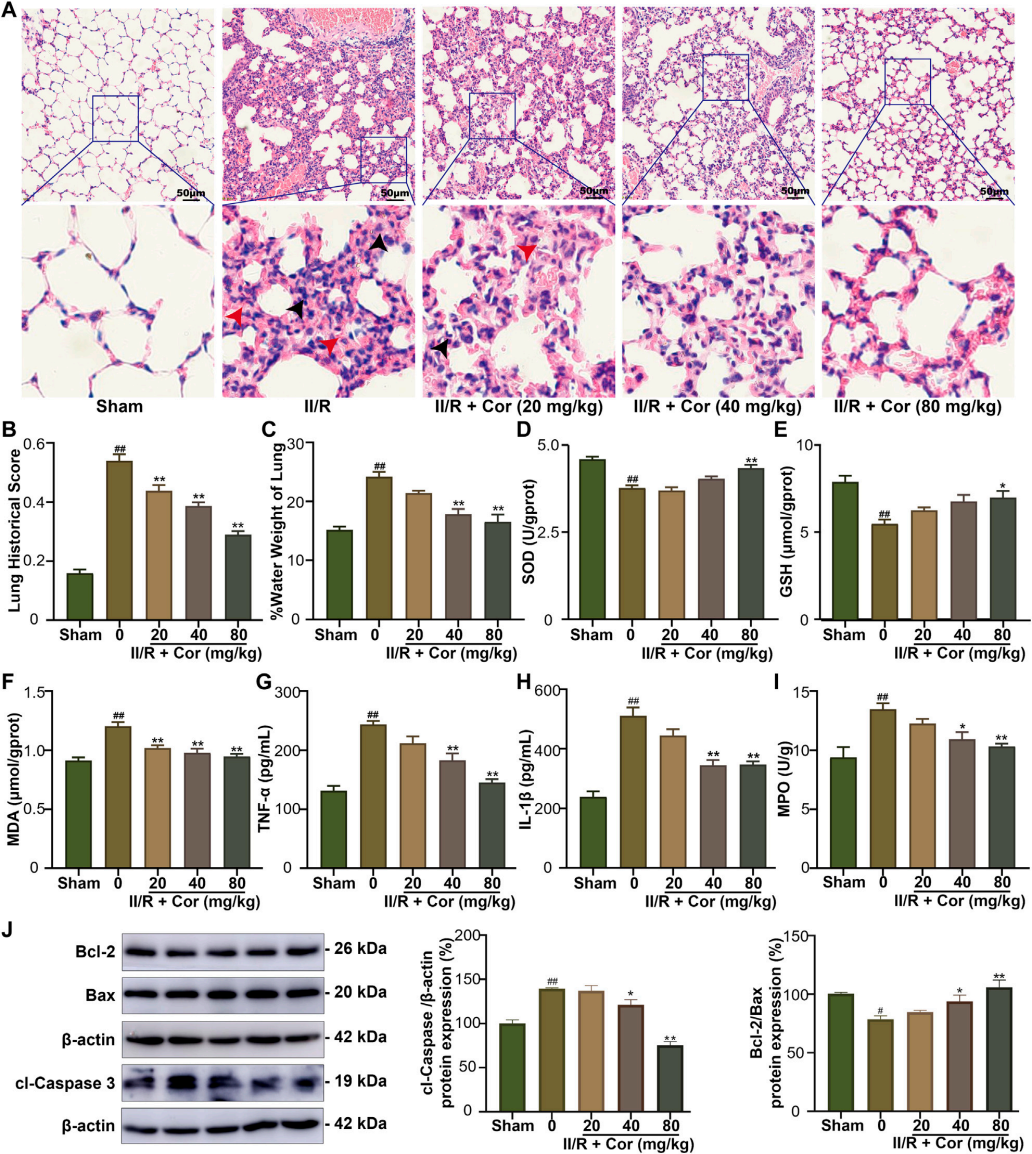
Cor ameliorated II/R-induced lung damage in mice. **(A)** Representative images of lung histology (scale bar = 50 μm). **(B)** The lung histopathological score measured by the American Thoracic Society 2010 Scale for Acute Lung Injury in Laboratory Animals. **(C)** The water content in lung tissue. **(D–I)** Effects of Cor on the levels of SOD, GSH, MDA, TNF-α, IL-1β, and MPO in lung. **(J)** The protein expression levels of Bcl-2/Bax and cleaved caspase 3 in lung tissue by western blotting. Data are expressed as the mean ± SEM (*n* ≥ 3), ^#^ *p* < 0.05 and ^##^ *p* < 0.01 vs. sham group, *p < 0.05 and **p < 0.01 vs. II/R group.

II/R typically leads to secondary lung injury (Ito et al., 2020). To investigate whether Cor could alleviate II/R-induced lung injury, the lung histopathological damage and lung water content were detected. As shown in Figures 2A,B, unclear alveolar structure, enlarged alveolar septum, inflammatory cell infiltration (black arrows), hemorrhage, and hyaline membrane formation (red arrows) were observed in II/R group with lung histopathological damage scores significantly increased from 0.15 ± 0.01 to 0.53 ± 0.02 (*p* < 0.01 vs. sham group). Moreover, the lung water content ratio was significantly increased to 24.18% ± 0.83 in II/R mice compared with 15.19% ± 056 in sham group (*p* < 0.01). Cor pre-treatment (20, 40 and 80 mg/kg) significantly alleviated the lung histopathological damage and decreased the tissue score and lung water content ratio to 0.29 ± 0.01 and 16.55% ± 1.24 (*p* < 0.01 vs. II/R group), respectively (Figure 2C). These results suggested that Cor could alleviate II/R-induced lung histopathological damage and pulmonary edema.

Further, the changes of oxidative stress, inflammatory response, and apoptosis-related indicators were examined. Our results indicated that the SOD activity and GSH content were significantly decreased from 4.52 ± 0.07 to 3.71 ± 0.07 U/gprot (*p* < 0.01) and from 7.85 ± 0.34 to 5.47 ± 0.24 μmol/gprot (*p* < 0.01), respectively, and the MDA content was markedly increased from 0.91 ± 0.03 to 1.20 ± 0.04 μmol/gprot (*p* < 0.01) in II/R group compared with the sham mice. Cor pre-treatment (20, 40 and 80 mg/kg) not only reversed the decreased SOD activity and GSH content to 4.27 ± 0.10 U/gprot (*p* < 0.01) and 6.96 ± 0.37 μmol/gprot (*p* < 0.05), respectively, but also reversed the increased MDA content to 0.95 ± 0.02 (*p* < 0.01), as compared with II/R group (Figures 2D–F). The levels of TNF-α, IL-1β and MPO were examined to characterize the protective effects of Cor on II/R-induced lung inflammation. As a result, the pro-inflammatory cytokines (IL-1β and TNF-α) and MPO activity were significantly increased in II/R-injured lung tissues compared with the sham group. Cor pre-treatment (20, 40 and 80 mg/kg) significantly and dose-dependently reduced these increased inflammatory cytokines from 243.83 ± 5.59 to 145.33 ± 5.82 pg/ml (TNF-α content, *p* < 0.01), from 510.67 ± 28.47 to 347.17 ± 11.04 pg/ml (IL-1β content, *p* < 0.01) and from 13.47 ± 0.50 to 10.32 ± 0.23 U/g tissue (MPO activity, *p* < 0.01), respectively (Figures 2G–I). Furthermore, the protein expression ratio of Bcl-2/Bax was decreased and protein expression level of cl-Caspase 3 was upregulated in II/R injured lung tissues, and Cor pre-treatment had significantly inhibited cell apoptosis in lung by reversing the II/R-induced dysregulation of Bcl-2/Bax ratio and cl-Caspase 3 (Figure 2J). Therefore, the above results indicated that Cor could ameliorate II/R-induced lung injury by inhibiting II/R-induced oxidative stress, inflammatory response, and apoptosis.

### Cor prevented II/R-induced NLRP3 inflammasome activation and pyroptosis in mice

**FIGURE 3:**
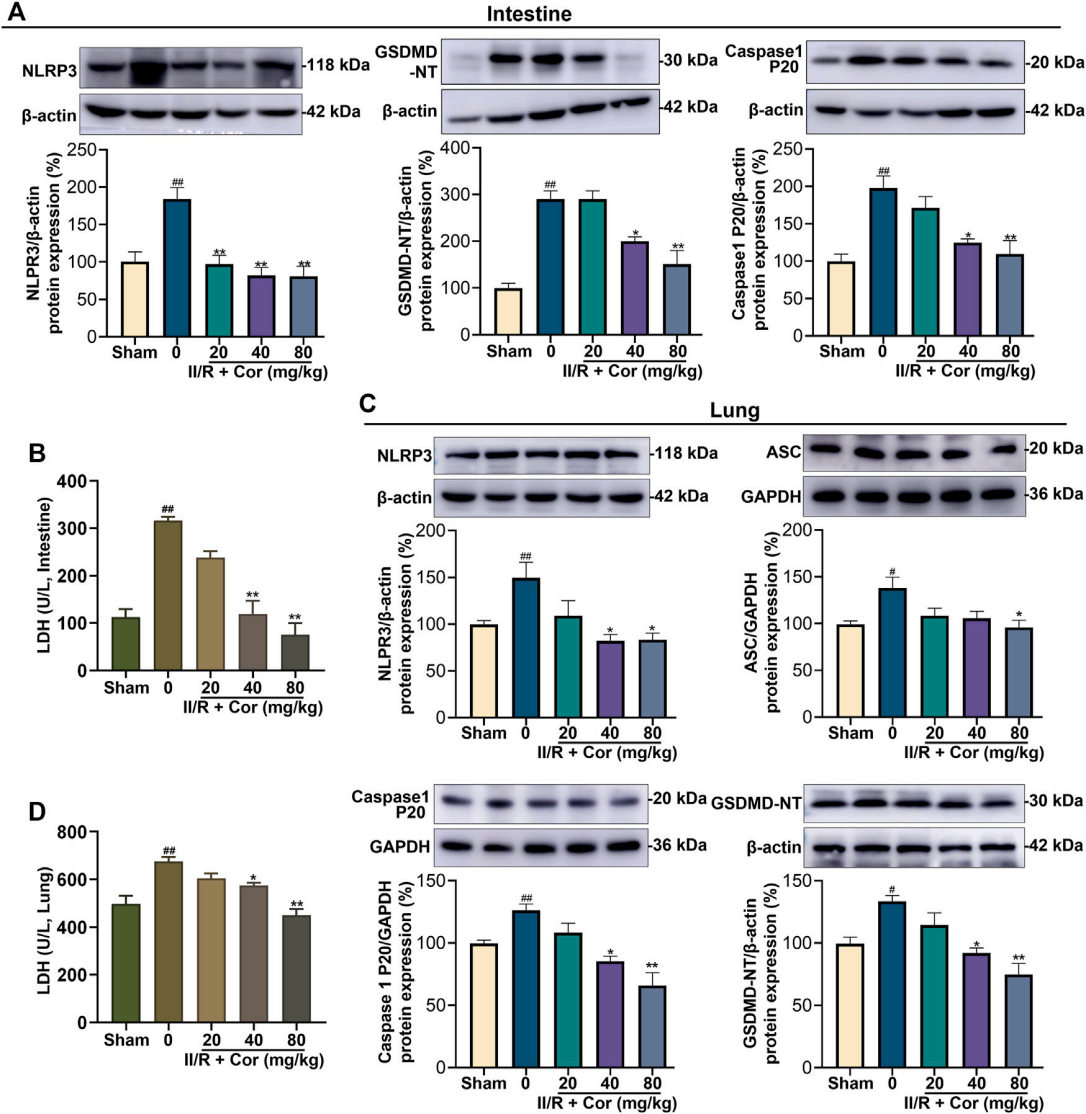
Cor inhibited NLRP3 inflammasome activation and alleviated pyroptosis in mice. **(A)** The protein expression levels of NLRP3, caspase 1 P20, and GSDMD-NT in intestine tissue. **(B)** The effects of Cor on LDH level in intestine tissue. **(C)** The protein expression levels of NLRP3, ASC, caspase 1 P20, and GSDMD-NT in lung tissue. **(D)** The effects of Cor on LDH level in lung tissue. Data are expressed as the mean ± SEM (*n* ≥ 3), ^#^ *p* < 0.05 and ^##^ *p* < 0.01 vs. sham group, *p < 0.05 and **p < 0.01 vs. II/R group.

The overactivation of NLRP3 can induce pyroptosis, accompanied by plasma membrane rupture and the over-release of inflammatory cytokines and LDH. We had detected the expression levels of NLRP3 and the markers of NLRP3 inflammasome activation, such as caspase-1 p20 subunit and the N-terminal fragment (GSDMD-NT) generated from the cleavage of pyroptosis execution protein GSDMD. As shown in Figure 3, II/R injury significantly up-regulated the expression levels of NLRP3, caspase-1 p20 and GSDMD-NT in both intestinal and lung, and Cor dose-dependently decreased the expression of NLRP3 protein and the release of caspase 1 p20 and GSDMD-NT, as compared with II/R group (Figures 3A,C, *p* < 0.05). Further, the LDH activities in both intestinal and lung tissues were markedly increased from 112.42 ± 16.62 to 316.05 ± 8.11 U/L (*p* < 0.01) and from 497.83 ± 33.10 to 674.85 ± 18.64 U/L (*p* < 0.01) after II/R injury, respectively. Cor pre-treatment (20, 40 and 80 mg/kg) inhibited II/R-induced LDH activity in a dose-dependent manner (Figures 3B,D). These results indicated that Cor prevented II/R-induced pyroptosis in both intestinal and lung tissues by suppressing NLRP3 inflammasome activation and GSDMD cleavage.

### Cor prevented oxygen-glucose deprivation/reoxygenation-induced NLRP3 inflammasome activation and pyroptosis *in vitro*

**FIGURE 4:**
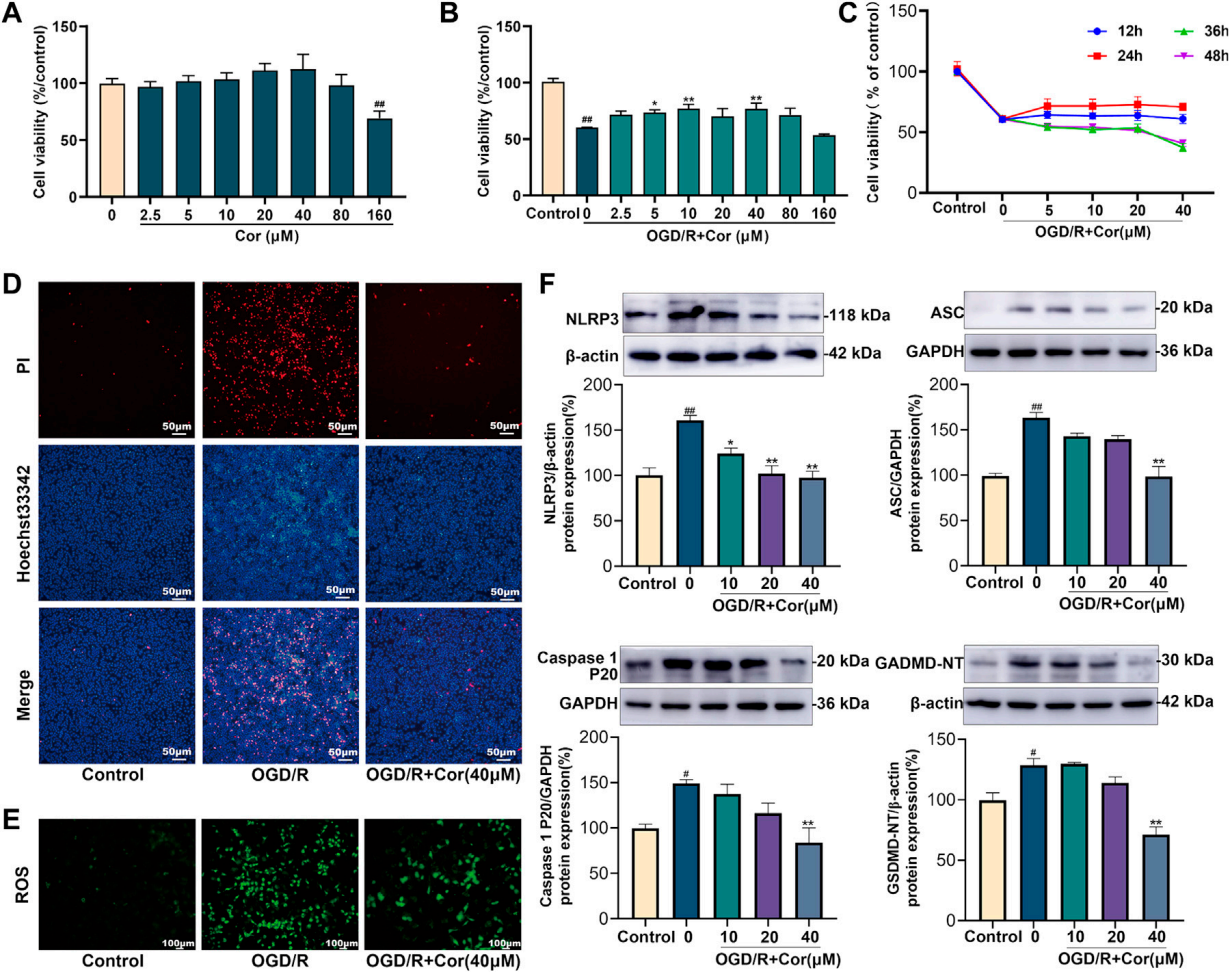
Cor inhibited NLRP3 inflammasome activation and alleviated pyroptosis in OGD/R injured MLE-12 cells. **(A)** Cytotoxicity of Cor on MLE-12 cells. **(B)** The protective effects of Cor on the OGD/R injured MLE-12 cells. **(C)** The effects of Cor on OGD/R injured MLE-12 cell with pre-treatment for different times. **(D)** Pyroptosis determination of MLE-12 cells using Hoechst 33342/PI double staining (scale bars = 50 μm). **(E)** The ROS determination of MLE-12 cells (scale bars = 100 μm). **(F)** The protein expression levels of NLRP3, ASC, caspase 1 P20, and GSDMD-NT in MLE-12 cells. Data are expressed as the mean ± SEM (*n* ≥ 3), ^#^ *p* < 0.05 and ^##^ *p* < 0.01 vs. sham group, *p < 0.05 and **p < 0.01 vs. OGD/R group.

An OGD/R model was established in IEC-6 and MLE-12 cells to mimic II/R-induced intestinal and lung injury *in vitro*. Cells were incubated in serum/glucose-free DMEM under a hypoxic condition to simulate ischemia by deprivation of oxygen and glucose. The CCK-8 assay showed that Cor, at a concentration of 80 μM or lower, did not exert observable influence on cell viability under routine conditions (Figure 4A). After OGD/R, the viability of MLE-12 cells was notably decreased, but Cor pre-treatment at concentrations of 10, 20, and 40 μM for 24 h produced the most significant restoration of cell viability than 12, 36 and 48 h administration. This result may be related to the metabolism of drugs in cells. Thus, 40 μM administration for 24 h was selected for the subsequent *in vitro* experiments (Figures 4B,C).

PI/Hoechst33342 double-staining assay was further applied to investigate the effects of Cor on pyroptosis in OGD/R-injured MLE-12 cells. PI-positive cells were markedly increased after OGD/R injury compared with the control group in which few PI-positive cells were observed. Cor (40 μM) pre-treatment reduced the percentage of PI-positive cells (Figure 4D). Cor also prevented the OGD/R-induced overproduction of reactive oxygen species (ROS; Figure 4E). In addition, western blotting results showed that the expression levels of NLRP3, ASC, mature caspase-1 and GSDMD-NT were all up-regulated upon OGD/R-injury, while Cor pre-treatment (10, 20 and 40 μM) significantly reversed the dysregulated expression of those proteins (Figure 4F), which was consistent with the *in vivo* results. Similarly, OGD/R-induced pyroptosis in IEC-6 cells was also alleviated by Cor through inhibiting NLRP3 inflammasome activation (Supplementary Figure S1). The results confirmed the anti-pyroptosis effects and the regulation of NLRP3 inflammasome activation by Cor during OGD/R injury in both lung and intestinal *in vitro*.

### Cor significantly inhibited the specific activation of NLRP3 inflammasome pathway

In OGD/R-injury IEC-6 cells, the increased percentage of PI-positive cells and up-regulated expression levels of NLRP3, mature caspase-1 and GSDMD-NT induced by OGD/R injury were significantly reversed by Cor pre-treatment (1 μM). However, this reversing effect of Cor was interrupted by Nigericin, an activator of NLRP3 (Supplementary Figure S1). These results suggested that Cor may alleviate II/R injury by preventing NLRP3 related pathways and the resulting pyroptosis.

**FIGURE 5:**
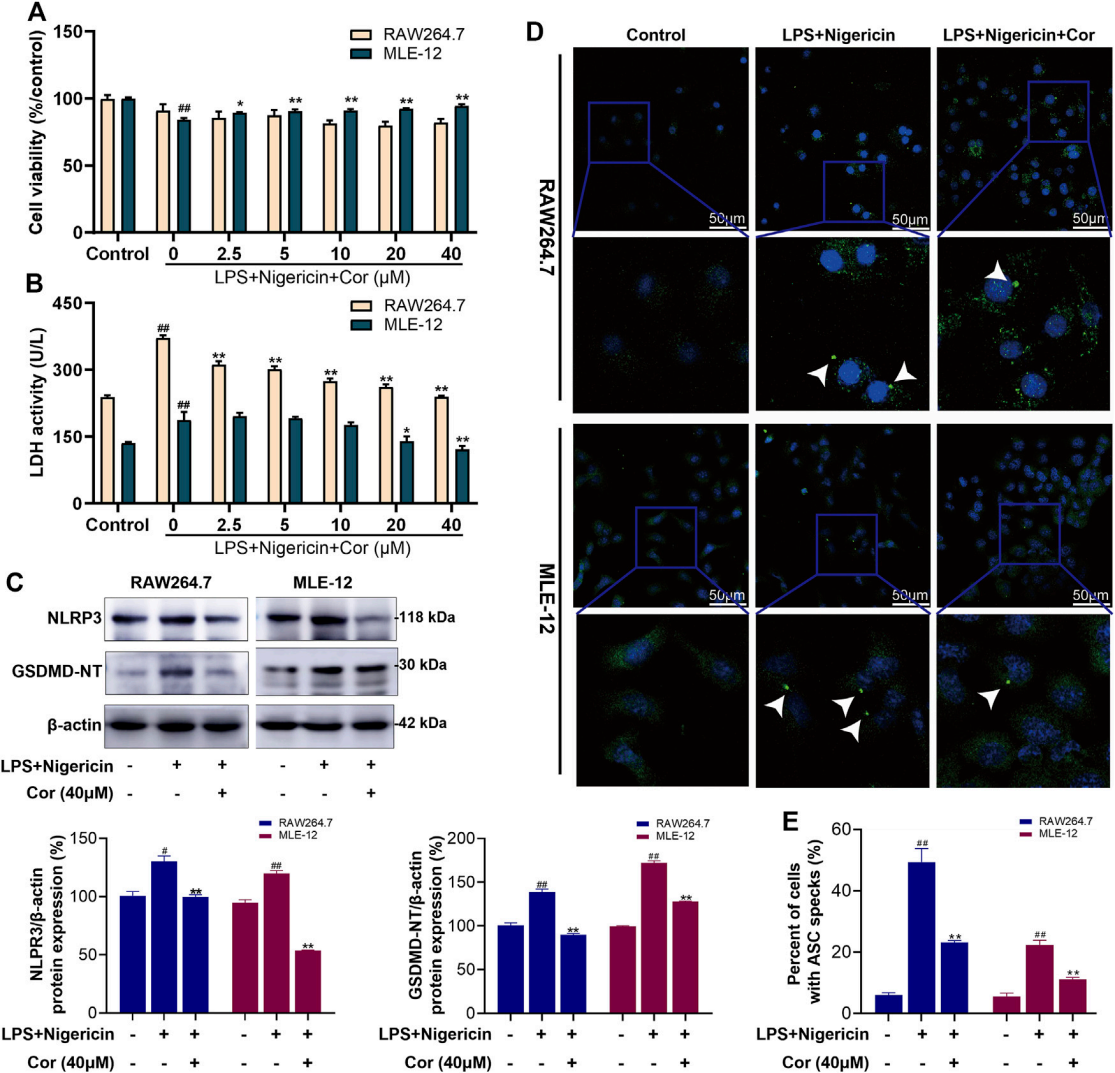
Cor inhibited the NLRP3 inflammasome pathway activated by LPS and Nigericin in RAW264.7/MLE-12 cells. **(A)** The effects of Cor on cell viability of LPS and nigericin treated RAW264.7 and MLE-12 cells. **(B)** The effects of Cor on LDH levels in LPS and nigericin treated RAW264.7 and MLE-12 cells. **(C)** The effects of Cor on the protein expression levels of NLRP3 and GSDMD-NT in LPS and nigericin treated RAW264.7 and MLE-12 cells. **(D,E)** The effects of Cor on the percentages of cells with ASC specks in LPS and nigericin treated RAW264.7 and MLE-12 cells. Data are expressed as the mean ± SEM (n≥3), ^#^ *p* < 0.05 and ^##^ *p* < 0.01 vs. sham group, *p < 0.05 and **p < 0.01 vs. LPS and nigericin treated group.

To further test whether Cor specifically inhibited the activation of NLRP3 inflammasome pathway, MLE-12 and RAW264.7 cells were incubated with LPS (100 ng/ml) + Nigericin (10 μM) to induce the specific activation of NLPR3 inflammasome pathway. For RAW264.7 cells, incubation with LPS and Nigericin did not cause significant changes in cell viability, but significantly increased the LDH activity from 239.1 ± 4.2 to 371.2 ± 6.2 U/L (Figures 5A,B). And the protein expression levels of NLPR3 and GSDMD-NT were also increased from 100% ± 3.40 to 130% ± 4.99 (*p* < 0.01) and from 100% ± 3.00 to 139% ± 3.42 (*p* < 0.01), respectively (Figure 5C). Meanwhile, the percentage of cells with ASC speck formation was increased from 6.00% ± 0.77 to 49.40% ± 4.39 (*p* < 0.01) (Figures 5D,E). The results showed the specific activation of NLRP3 inflammasome pathway and cell pyroptosis after incubation with LPS and Nigericin. Fortunately, pre-treatment with Cor (2.5, 5, 10, 20 and 40 μM) significantly reversed the dysregulated LDH activity in a dose-dependent manner to 239.6 ± 2.9 U/L at 40 μM (Figure 5B). Moreover, pre-treatment with Cor (40 μM) significantly prevented the up-regulated protein expressions of NLRP3, GSDMD-NT and ASC (Figures 5C–E), indicating the inhibiting effect of Cor on the specific activation of NLRP3 inflammasome pathway and the resulting pyroptosis in RAW264.7 cells.

Similarly, the prevention effects of Cor on NLRP3 inflammasome pathway and pyroptosis were also observed in LPS and Nigericin incubated MLE-12 cells (Figures 5A–E), which further confirmed our speculation.

## Discussions

II/R is a clinical emergency that commonly occurs in several clinical conditions. In addition to causing local intestinal damage, II/R is often followed by distant organ injury, especially lung injury, which is associated with high morbidity and mortality. The development of innovative therapeutic strategies to mitigate II/R-induced intestinal and lung damage is of great significance.

**FIGURE 6:**
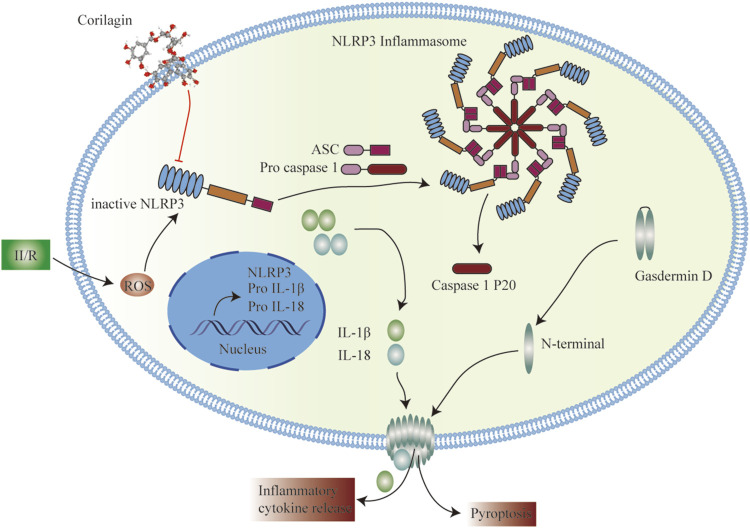
Schematic representation of the potential protective mechanism of Cor on II/R-induced injury through inhibiting the NLRP3 inflammasome pathway.

Cor, an ellagitannin, is a major active component of many ethnopharmacological plants such as *Phyllanthus niruri* L., P*. emblica* L. and *P. urinaria* L. Cor is reported to possess excellent pharmacological activities, including antioxidant, anti-inflammatory, hepatoprotective, and anti-tumor activities (Li et al., 2018). Recent studies have shown that Cor can protect against rat cerebral I/R injury by attenuating oxidative stress and enhancing angiogenesis and can improve I/R-induced acute lung injury by ameliorating the apoptosis pathway (Ding et al., 2017; Guo et al., 2017). The results of the current study indicated that Cor protects against II/R-induced intestinal and lung injury *via* inhibiting NLRP3 inflammasome activation and cell pyroptosis (Figure 6).

Oxidative stress is considered a crucial factor contributing to I/R-induced injury. Hypoxia leads to impaired mitochondrial oxidative phosphorylation, resulting in adenosine triphosphate (ATP) depletion and inhibition of the ATPase-dependent ion pump, which further degrades ATP to hypoxanthine. Simultaneously, available oxygen generates oxidative hypoxanthine and superoxide anion radicals (O^2−^) during reperfusion, which are converted to hydrogen peroxide (H_2_O_2_) and cleaved to hydroxyl radicals (HO^−^) (Bertoni et al., 2018). The ROS (O^2−^, H_2_O_2_ and HO^−^) triggers nucleic acid fragmentation and lipid peroxidation, leading to various cellular death and changes in microvascular and mucosal permeability (Holanda et al., 2021). In the current study, Cor significantly inhibited intestinal MDA content and up-regulated SOD activity, and similarly reversed the dysregulation of MDA content, SOD and GSH activities in lung tissue in the II/R mice, which is consistent with previous reports (Lv et al., 2019; Zhang et al., 2019). In addition, inflammation and cell death play significant roles in the progression of lung injury caused by various factors, including I/R and shock. The MPO activity and pro-inflammatory factors contents (TNF-α, IL-1β) in II/R mice lung tissues were significantly prevented by Cor, alleviating neutrophil aggregation and pulmonary edema, and Cor also reversed the apoptosis-associated protein Bcl2/Bax, cl-caspase3 protein expression.

Oxidative stress leads to an unfolded protein response that can cause detachment of thioredoxin from thioredoxin-interacting protein, which interacts with NLRP3 to promote activation of NLRP3 inflammasome (Toldo et al., 2018). NLRP3 is a recognition receptor that senses pathogen- and host-derived damage-associated molecular patterns to trigger the formation of NLRP3 inflammasome complex. The assembly of NLRP3 inflammasome complex activates pyroptosis *via* GSDMD-NT, eventually resulting in the release of inflammatory factors and LDH (Frank and Vince, 2019). Pyroptosis is reported to be involved in the development of I/R injury and studies have revealed that the intestinal barrier can be maintained by inhibiting the activation of NLRP3 inflammasome (Jun et al., 2019; Yun et al., 2021). In the current study, II/R-induced LDH release and activation of NLRP3 signaling in intestinal and lung tissues were inhibited by Cor. ROS stimulation enhanced NLRP3 inflammasome aggregation to increase pulmonary vascular permeability and inflammatory response, leading to vascular and interstitial pulmonary edema (Ito et al., 2020). Moreover, Cor prevented OGD/R-induced PI-positive cells and NLRP3 inflammasome signaling in IEC-6 and MLE-12 cells. On the other hand, Cor significantly prevented OGD/R-induced ROS accumulation in MLE-12 cells. We speculate that the protective effect of Cor is related to ROS. The above results suggest that pyroptosis participates in the development of II/R-induced intestinal and distal lung injury, and that Cor can alleviate pyroptosis.

ASC aggregates into dimeric molecules, known as ASC specks. ASC specks are dynamic structures that can amplify the NLRP3 response to weak stimuli by facilitating the formation of NLRP3 inflammasome, which, in turn, activate caspase-1 (Zhao et al., 2021b). Caspase-1 activity negatively regulates the frequency and size of ASC specks; however, caspase-1 is likely to be activated after leaving the present speck structure (Nagar et al., 2021). In RAW264.7 and MLE-12 cells, co-incubation with LPS and Nigericin caused an increase in NLRP3/GSDMD-NT expression, LDH leakage and ASC specks production, while Cor suppressed the above phenomena. In OGD/R-induced IEC-6 cells, the protective effect of Nigericin pre-treatment followed by Cor administration was diminished compared to Cor administration alone. This suggests that Cor prevents NLRP3 oligomerization or ASC speck production, thereby inhibiting the activity of the pyroptosis driver protein caspase-1 to alleviate pyroptosis.

The active ingredients from traditional ethnic herbal medicines often have multiple targets and involve multiple pathways (Fei et al., 2021). Previous studies have found that Cor can inhibit the activation of NF-κB pathway in a STAT3-associated manner, thereby downregulating inflammatory cytokine expression, and can regulate the immune response and relieve inflammatory injury by interfering with the TLR3 signaling pathway (Tong et al., 2016; Li et al., 2019). Thus, the specific mechanism by which Cor inhibits NLRP3 inflammasome to alleviate pyroptosis needs to be further elucidated. This may involve regulation by several transcription factors. Moreover, the exact impact of NLRP3 inflammasome assembly under Cor treatment also requires further investigation. The therapeutic effect of Cor may be attributed to the inhibition of NLRP3 signaling-associated pyroptosis. While this study possesses several strengths, there are also some limitations that should be noted. Specifically, the OGD/R model, as used in this study, cannot fully simulate the I/R environment due to the presence of amino acids and other components that would not be present in an ischemic event. Further, the interaction between Cor and the NLRP3 inflammasome was not clearly studied. Finally, the multiple targets of Cor require further investigation.

In summary, Cor alleviated II/R injury and distal lung injury. The therapeutic effects may be attributed to the prevention of NLRP3-associated pyroptosis. This study increases the drug selectivity for II/R treatment and provides a scientific reference for the standardized application of Cor in clinical practice. In the future, we will explore and illustrate the multiple targets of Cor, which will enable full exploitation of Cor for clinical applications.

## Data Availability

The raw data supporting the conclusion of this article will be made available by the authors, without undue reservation.
