# Supplementary material for: Corilagin alleviates intestinal ischemia/reperfusion-induced intestinal and lung injury in mice via inhibiting NLRP3 inflammasome activation and pyroptosis
Source: Front Pharmacol. 2022 Nov 23;13:1060104. doi: 10.3389/fphar.2022.1060104 (PMC9727192; doi:10.3389/fphar.2022.1060104)
Supplement: Supplementary file 1 [file DataSheet1.docx]

Supplementary Material

## Supplementary Figures

**
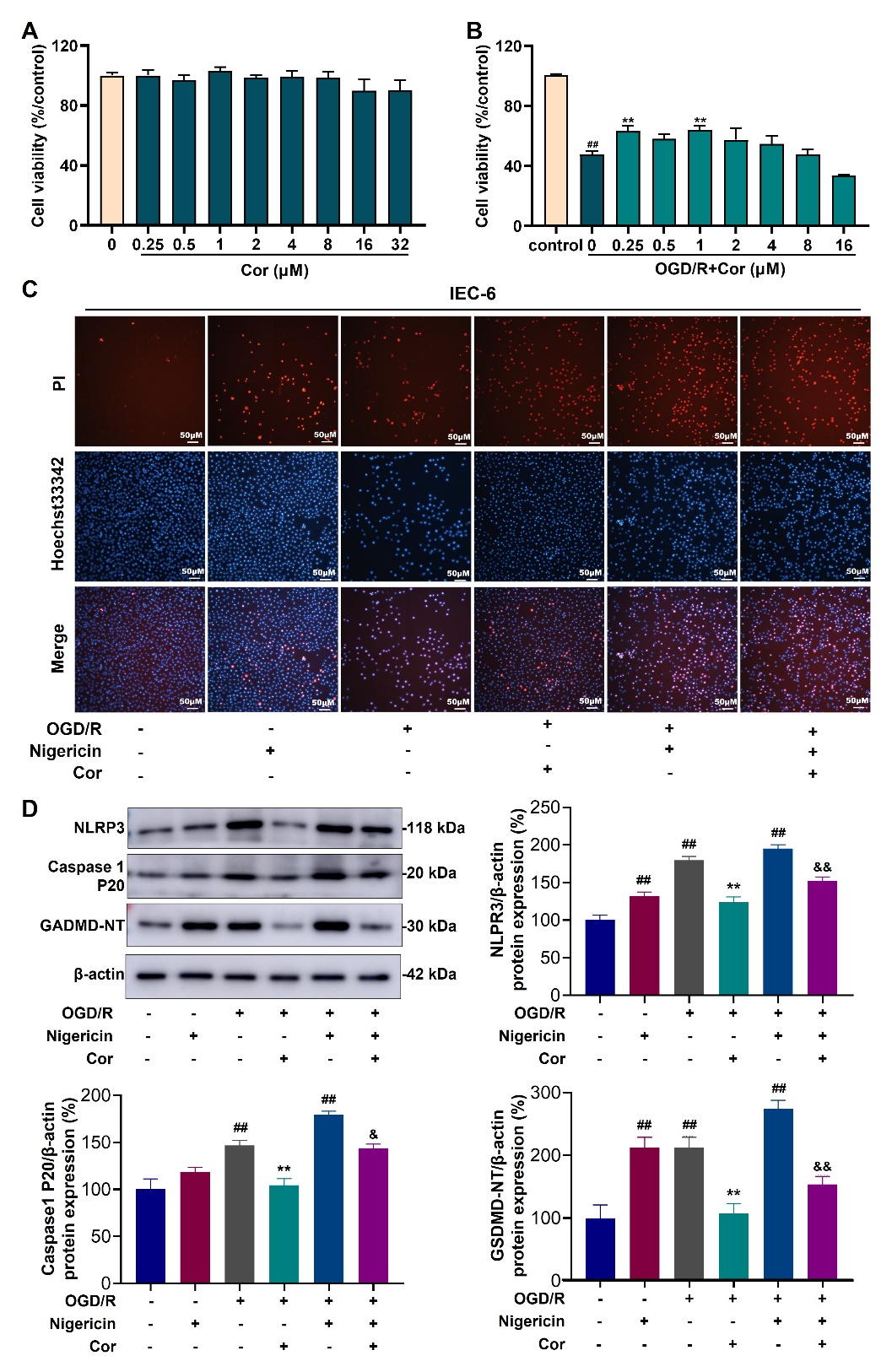
**

**Supplementary Figure S1.** **Cor inhibited the NLRP3 inflammasome pathway and pyroptosis in OGD/R injured IEC-6 cells.** (A) Cytotoxicity of Cor on IEC-6 cells. (B) The protective effects of Cor on OGD/R injured IEC-6 cells. (C) Pyroptosis determination of IEC-6 cells using Hoechst 33342/PI double staining (scale bars = 50 μm). (D) The protein expression levels of NLRP3, caspase 1 P20, and GSDMD-NT in IEC-6 cells. Data are expressed as the mean ± SEM (n≥3), ^##^*p* < 0.01 vs sham group, **p* < 0.05 and ***p* < 0.01 vs OGD/R group.
